# NMDA Receptor Model of Antipsychotic Drug-Induced Hypofrontality

**DOI:** 10.3390/ijms20061442

**Published:** 2019-03-21

**Authors:** Marek Krzystanek, Artur Pałasz

**Affiliations:** 1 Department and Clinic of Psychiatric Rehabilitation, Department of Psychiatry and Psychotherapy, School of Medicine in Katowice, Ziołowa 45/47, 40-635 Katowice, Poland; 2 Department of Histology, School of Medicine in Katowice, Medyków 18, 40-752 Katowice, Poland; artiassone@gmail.com

**Keywords:** hypofrontality, NMDA receptor, schizophrenia, antipsychotic drugs

## Abstract

Schizophrenia is a chronic mental disease, affecting around 1% of the general population. Schizophrenia is characterized by productive, negative, affective, and disorganization symptoms, and cognitive deficits. Cognitive deficits prevail in most of the schizophrenia patients and are one of the most disabling symptoms. They usually occur before the acute episode of the disease and tend to become chronic with no satisfactory treatment from antipsychotic drugs. Because of their early manifestation in patients’ lives, cognitive deficits are suggested to be the primary symptom of schizophrenia. The pathogenesis of cognitive deficits in schizophrenia is not fully understood. They are linked with hypofrontality, which is a decrease in blood flow and glucose metabolism in the prefrontal lobe of schizophrenia-suffering patients. Hypofrontality is linked with disturbances of the corticolimbothalamic circuit, important for cognition and memory in humans. The circuit consists of a group of neuroanatomic structures and hypothetically any disturbance in them may result in cognitive deficits. We present a translational preclinical model of understanding how antipsychotic medication may decrease the N-methyl-D-aspartic acid (NMDA) receptors’ activity and produce dysfunctions in the corticolimbothalamic circuit and hypofrontality. From several pharmacological experiments on rats, including mainly our own recent findings, we collected data that suggest that antipsychotic medication may maintain and escalate hypofrontality in schizophrenia, decreasing NMDA receptor activity in the corticolimbothalamic circuit in the human brain. We discuss our findings within the literature of the subject.

## 1. Introduction

Schizophrenia is a mental disease that affects circa 1% of the general population [1]. Schizophrenia seriously impairs several aspects of everyday life, such as interpersonal relationships or professional activity, often limiting a patient’s independence [2]. This mental disease has a chronic character and is characterized by multidimensional symptomatology in the form of productive, negative, affective, and disorganization symptoms, and cognitive deficits.

Cognitive deficits in schizophrenia are frequent and persistent, prevalent in 60–70% of patients [3]. Persistent disability in schizophrenia results mainly from cognitive disturbances and negative symptoms [4,5,6]. Chronic persistence of cognitive disturbances in schizophrenia is the worst prognostic factor for a patient’s social abilities [7]. In turn, an improvement of cognitive functioning is related to better social functioning [8].

Many long-term studies indicate the cognitive dysfunctions in schizophrenia are present at an early stage of the disease, even before the acute phase develops, and then they become chronic with no complete improvement in the coming years [9,10,11,12,13,14,15]. Cognitive deficits start in most patients before the first schizophrenic episode and are not present in childhood [16]. For this reason, cognitive disturbances are understood to be the primary symptoms of schizophrenia [17].

Cognitive deficits are linked with so-called hypofrontality. Hypofrontality is the reduced utilization of glucose and decreased blood flow in the prefrontal cortex, that implies the decrease of neuronal metabolism and impaired cognition [18]. The concept of hypofrontality is broadly described in the literature of schizophrenia, although the phenomenon is not fully understood and the strict neuronal mechanism of it is not known. It is supposed that hypofrontality relates to impairment of neuronal transmission and synaptic connectivity, that are based on genetic predisposition and neurodevelopmental disturbances [18]. 

Identification of mechanisms of how cognitive impairment develops in schizophrenia and finding its effective treatment is crucial for the improvement of health and functioning of patients with schizophrenia. On a theoretical basis, the glutamatergic N-methyl-D-aspartic acid receptors (NMDARs) play an important role in the mechanism of development and maintenance of cognitive deficits in schizophrenia. It is known that cognitive deficits in schizophrenia relate to disturbances of transmission in the corticolimbothalamic circuit [18]. The transmission in that loop is regulated by NMDARs on GABA-ergic inhibitory interneurons. Besides that, NMDARs in synapses play their essential role in neuronal connectivity. 

We designed a series of experimental studies to evaluate the expression of NMDAR subunits in structures belonging to the corticolimbothalamic circuit. They aimed to elucidate how antipsychotic drugs, used in the treatment of schizophrenia, modulate the NMDAR composition, and indirectly, how they influence the receptor activity and the activity of the glutamatergic system. 

## 2. Glutamatergic Signaling and Cognitive Deficits in Schizophrenia

### 2.1. An Outline NMDA Receptor Structure and Function

**Figure 1:**
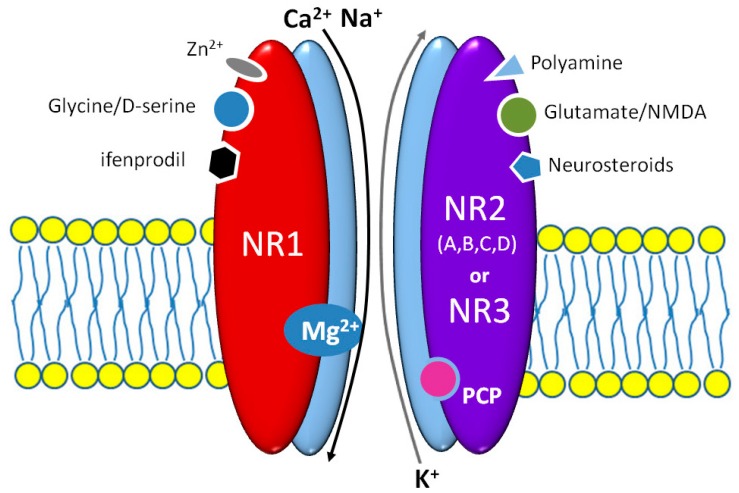
The scheme of N-methyl-D-aspartic acid (NMDA) receptor structure with its main binding sites in the context of an agonistic/antagonistic influence of the most important native and exogenous ligands.

Brain NMDARs determine the existence of higher mental functions playing a fundamental role in the mechanisms of consciousness, memory, learning, emotions, and even in some motor functions. NMDAR molecules are heteromers consisting of two obligatory NR1 and two or three NR2 subunits, as shown in Figure 1 [19]. Several isoforms of NR1 and at least four classes of NR2 subunit—A, B, C, and D—were identified. Diverse NR2A classes may differentially affect NMDAR activity and its modulation by both agonists and antagonists. The NR1 subunit does expose the lifelong expression in almost all brain structures [20]. NR1, NR2A, and NR2B subunits are also widely distributed in the central nervous system (CNS), especially in the hippocampus, thalamus, and prefrontal cortex, while the NR2C and NR2D ones are preferentially expressed in the cerebellum, brainstem, and spinal cord. It appears that pharmacological and biophysical activity of NMDARs is determined mainly by NR2 subunits [21]. Several regulatory domains of the NR2 subunit can bind numerous endo- and exogenous factors, including drugs e.g., polyamines, protons, zinc ions, glutathione, neurosteroids, ifenprodil, eliprodil, or even haloperidol [19]. All NMDAR subunits are strictly structurally and functionally connected, therefore modulatory sites of NR2 affect the glycine/D-serine binding site of NR1 [22]. The NR3 subunit is detected in the CNS only in the prenatal period and early childhood. NR3 suppresses the NMDAR activation when the receptor molecule consists of NR1 and NR2 subunits. An integration on NR3 merely with NR1 subunits may cause the origin of glutamate insensitive NMDARs [23]. This kind of NMDAR that may play a role in the pathogenesis of schizophrenia occurs during brain development; however, their evidence in adults is not so far confirmed [24]. Glycine and glutamate binding sites are located in the homologous domains of NR1 and NR2A/B subunits, respectively [25]. Despite their separate location, both sites are strictly functionally connected. Glycine binding facilitates glutamate ligation, extends the channel opening, and delays NMDAR desensitization [26]. Most importantly, the glutamate binding is not enough for NMDAR activation; a simultaneous postsynaptic membrane depolarization to remove Mg^2+^ from the channel pore and glycine or D-serine binding are strictly required [27]. NMDARs contain a nonselective cation channel, that enable Ca^2+^ and Na^+^ influx into the neuron. An increased calcium concentration in the neuroplasm causes activation of adenylate cyclase, Ca^2+^, and calmodulin-dependent protein kinase II (CaMKII). A common property of all NMDARs is binding several, psychomimetic open channel blockers (OCB)—ketamine, esketamine, phencyclidine (PCP), and dizocilpine [26].

### 2.2. NMDA Receptor-Related Glutamatergic Transmission Impairment in Schizophrenia

One of the hypotheses of how cognitive deficits develop in schizophrenia is a pharmacological model of NMDAR insufficiency. The model explains the acute and chronic consequences of NMDAR insufficiency for acute symptoms of schizophrenia and hypofrontality. In this model, two clinical situations are reflected: an acute schizophrenic episode and a chronic stage of the disease [18]. The pharmacological model of NMDAR insufficiency shows that both hyper- and hypoglutamatergic states lead to pathologic results in the human brain. 

An acute receptor antagonist model refers to the short time NMDAR blockade with antagonist phencyclidine (PCP). The blockade of NMDAR on GABA interneurons results in glutamatergic disinhibition in the prefrontal cortex (hyperfrontality) [28]. Neurochemical disturbances, present in the human brain in the acute phase of schizophrenia, resemble the changes observed when NMDAR antagonists are administered directly into different areas of the brain [29]. For example, a significant increase of glutamatergic activity in the cingulate gyrus was observed both in patients with early psychosis [30] and in its prodromal phase [31]. In animal models, a single dose of PCP or ketamine, the dissociative anesthetics, and NMDAR ionic channel blockers, induces neurotoxicity and as a consequence, a degeneration of neurons in many rat brain regions [32]. Finally, an administration of ketamine to healthy volunteers induces an increase of glutamine excretion in the brain [32]. It is worth mentioning that both ketamine and esketamine, as well as newest the NMDAR inhibitors, e.g., rapastinel or apimostinel, are recently considered as potentially promising factors in the therapy of treatment-resistant depression and mood disorders [33,34].

The chronic PCP model explains the link between NMDAR insufficiency and hypofrontality [18]. Long-term administration of PCP inhibits glucose metabolism in rat prefrontal cortex and decreases expression of glutamate decarboxylase—the protein marker of GABA interneuron activity. It is also suggested that a decrease of cortical GAD67 expression in schizophrenia can impair neuroplasticity and can be involved in the hypofrontality arising [35]. NMDARs play a crucial role both in the origin and function of brain GABA-ergic interneurons, especially chandelier and basket cells [36], being responsible for all behavioral effects occurred after application of their modulators [37]. A unique and crucial role is currently ascribed to GABA-ergic parvalbumin neurons [38]. A decreased parvalbumin expression in dorsolateral prefrontal cortex and abnormal gamma band oscillations in schizophrenic patients may be connected with GABA-ergic neurons impairment [39,40]. Furthermore, the NMDAR hypofunction in the cortical neurons can also trigger overstimulation of GABA-ergic cells in the central tegmental area, disturb the mesocortical dopaminergic pathway activity, and cause negative and cognitive symptoms of schizophrenia [41]. The expression of serotonin receptor 5-HT_1A_ in the prefrontal cortex is increased but 5-HT_2A_ is reduced, and that may be associated with the pathogenesis of emotional disturbances typical for schizophrenia [42].

In schizophrenia-suffering patients, the same as in people abusing PCP, symptoms of hypofrontality and GABA receptor deficiency are observed. It was shown that the hypofrontality symptoms are maintained even if GABA receptor functioning is restored by clozapine [18]. In the acute phase of schizophrenia, NMDAR insufficiency on GABA-ergic interneurons disinhibits glutamatergic, serotoninergic, and dopaminergic transmission (hyperfrontality). As a consequence, a down-regulation of receptors occurs and leads to a decrease of dopamine and glutamine turn-over in the prefrontal lobe and to deficit symptoms (hypofrontality), corresponding with a chronic phase of psychosis [7,43]. Presumably, the repetitive or long-term hyperglutamatergic states induce receptors’ adaptation and result in a decrease of neurotransmission in the prefrontal cortex [7]. It should be underlined that some primary functional dysregulations of NMDA receptors may cause monoamine signaling disturbances [44]. Patients suffering from schizophrenia also show significant NMDAR related deficits of neuroplasticity in the auditory cortex with a decrease of cognition. The deficits can be partially reduced with NMDAR agonist D-serine [45].

A piece of interesting information about hypofrontality may be derived from studies on rats prenatally exposed to methylazoxymenthanol (MAM). This is a genotoxic agent, that methylates DNA helix and impairs neuronal differentiation and migration. In these animals, some structural mPFC dysfunctions, as well as several schizophrenia-like behavioral abnormalities, were observed [46,47]. A recent magnetic resonance imaging (MRI) and diffusion tensor imaging (DTI) finding on MAM rats proved that hypofrontality observed in young individuals is maintained in adult ones being associated with hyperflexible phenotype and posterior hyperactivity [48]. These novel kinds of basic, noninvasive imaging studies can be clinically translatable and contribute significantly to the development of the early diagnosis of schizophrenia.

Several studies confirm the reliability of the long-term PCP administration model. Long-term exposition of rats to NMDAR antagonist decreases brain activity [48] and decreases the dopamine release in the prefrontal cortex and limbic system [49]. Similarly, in humans without schizophrenia, the long-term exposition to PCP decreases the glucose metabolism in the prefrontal cortex [29]. Long-term PCP administration is a cause of apoptosis in the brain cortex. It was confirmed that the apoptosis is related more to the blockade of NMDARs, containing more NR2A than NR2B subunits [50].

In schizophrenia patients, post mortem studies showed changes in the composition of NMDAR subunits in different human brain areas. Despite the fact that the results are frequently inconsistent, they indicate a decrease of NMDAR activity in schizophrenia [27]. Based on the studies, some changes of NMDAR subunit expression in schizophrenia can be characterized. Namely, in people with schizophrenia in the dorsolateral prefrontal cortex, expression of NR1 and NR2A subunits (both mRNA and protein) is decreased [51,52,53]. On the other hand, no changes in cortex expression of NR2 (A, B, and D) and NR3 are observed, except a decrease of NR2C subunit expression in the prefrontal lobe [51,54]. The transport of the NR2B subunit in the cortex can be disturbed too, so in that way, the activity of NMDARs can be decreased [55]. In the frontal and occipital cortex, NR2A subunit expression is increased [19], while in the parietal cortex the quantity of NR2B subunits increases [56]. In the hippocampus, NR1 expression decreases, but probably more NR2B subunits are synthesized [57]. In the thalamus NR1 subunits are less expressed [19] and in the dorsomedial nucleus the number of NR2B subunits increases [58]. In the putamen the number of NR1 subunits decreases [59]. Because the NR1 subunit is present in each NMDAR, so the decrease of it indicates the decrease of the receptor expression in the striatum. Similarly, in the substantia nigra the number of NR1 subunits is decreased [19]. Still, parts of some studies do not show any abnormalities, e.g., [56]. Some patients suffering from the initial phase of NMDAR autoimmune encephalitis with NR1 antibodies may exhibit schizophrenia-like psychotic behavior [60,61] or symptoms of hypofrontality [62], however, the vast majority of recent studies do not confirm the presence of IgG-NR1 antibodies in schizophrenia [63,64].

Concluding, the expression of the NR1 subunit of NMDARs in most structures of the brain in schizophrenia seems decreased and NR2B subunit production is increased. However, the interpretation of the findings should be cautious. The material comes from people suffering from schizophrenia and treated chronically with antipsychotics. The findings may be both the result of the pathogenesis of schizophrenia and the influence of the pharmacological treatment. It is known that antipsychotics may change the expression of genes for NMDAR subunits and thus change the receptor functioning [65].

The treatment of the disturbances of glutamatergic transmission in schizophrenia should be focused on rebuilding the balance between the inhibition and stimulation in neurotransmission systems in the CNS [5] by decreasing the glutamatergic activity in the acute phase and its increase when symptoms of hypofrontality occur. The goal can be achieved both with a change of a number of NMDARs, receptor subunit composition, or receptor activity.

In the present work, we compared and concluded results from a series of our own studies, aimed at analyzing the influence of antipsychotics on the expression of NMDAR subunits in the rat brain. We performed immunohistochemical studies of the influence of four-week intraperitoneal administration of olanzapine, clozapine, or haloperidol on the expression of NMDAR subunits: NR1, NR2A, and NR2B in CA1, CA2, and CA3 hippocampal regions, gyrus dentatus, neocortex, thalamus, and in subthalamus [66,67]. Obtained results show that the expression of NMDAR subunits has different modality, depending on the brain area and the used antipsychotic.

We propose a model of how antipsychotics change the composition of NMDARs and its hypothetical consequences for hypofrontality. That model can be helpful in understanding the mechanisms of hypofrontality induced by antipsychotics.

## 3. Expression of NMDA Receptor Subunits in the Rat Brain after Antipsychotic Treatment

### 3.1. Hippocampal Formation

**Figure 2:**
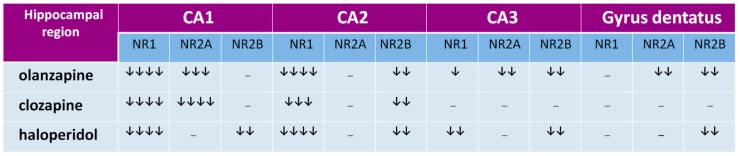
The influence of chronic antipsychotic drug administration on NMDA receptor subunits’ expression in CA1, CA2, and CA3 hippocampus regions and in gyrus dentatus in rats. Arrows indicate the subunit expression decrease by 0–25% (↓), 26–50% (↓↓), 51–75% (↓↓↓), and 76–100% (↓↓↓↓).

**Figure 3:**
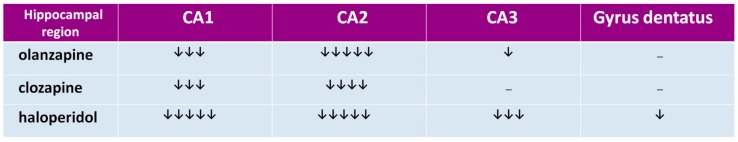
The influence of chronic antipsychotic drug administration on glutamatergic activity in CA1, CA2, and CA3 hippocampus regions and in gyrus dentatus in rats. A number of arrows indicate the neuroleptic’s potential to decrease the glutamatergic activity, resulting from the summation of NR1 expression, and proportion of NR2A to NR2B expression (an arrow was added when NR2B expression prevailed, and subtracted when NR2A expression prevailed).

The hippocampal formation, a part of the *archicortex*, plays a key role in the most widely understood cognitive mechanisms [68,69,70] and its numerous dysfunctions are often described in schizophrenia [71,72,73,74]. In hippocampal regions CA1 and CA2, all tested drugs decrease expression of the NR1 subunit, that indicates that olanzapine, clozapine, and haloperidol decrease the number of NMDARs in those structures, as shown in Figure 2 [66]. In the CA3 area, haloperidol and olanzapine but not clozapine, decrease NMDAR numbers, as shown in Figure 2. The data suggest that chronic administration of antipsychotics can decrease the glutamatergic activity in the hippocampus, as shown in Figure 3. Hanaoka et al. (2003) in a similar study found that both haloperidol and clozapine given to rats for two weeks decrease expression of the NR2B subunit in the hippocampus [75]. In that study, results were the same after four weeks, which indicates the durability of the changes of NR2B subunit expression in long-term drug administration. Schmitt et al. (2003) showed that both haloperidol and clozapine, given for 6 months, reduce expression of NR2A subunits in the CA1 hippocampal region [76]. In the study by Riva et al. (1997), one dose of haloperidol evoked an increase of expression of mRNA for subunits NR2A and NR2B, but the effect withdrew after 21 days of continuous administration of the antipsychotic [77].

**Figure 4:**
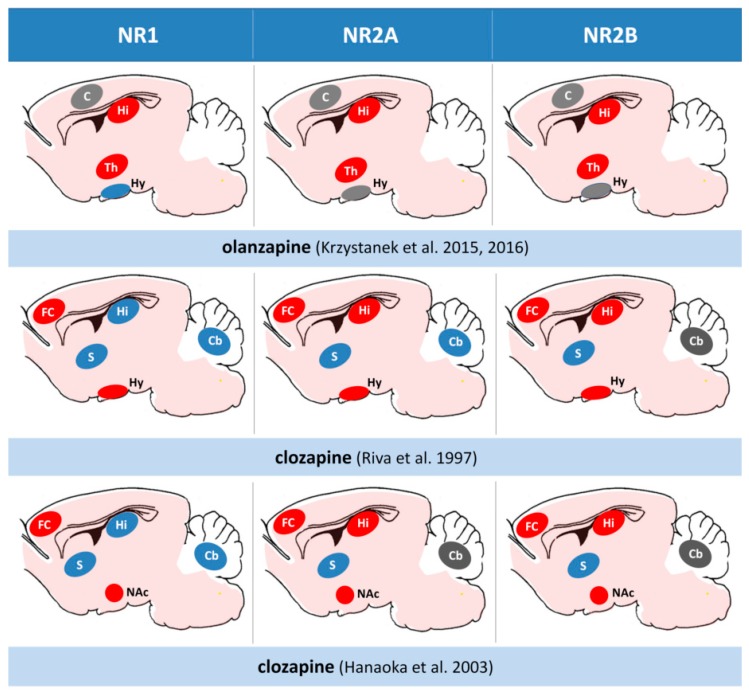
A comparative effect of second-generation antipsychotics on the NMDA receptor subunits expression in the rat brain. A comparative juxtaposition of the main studies in the field depicting brain structures with decreased (red) or increased (blue) NMDR subunit expression. Changes statistically not significant or no data in gray. Hi; hippocampus, FC; frontal cortex, PC; parietal cortex, Th; thalamus, Hy; hypothalamus, S; striatum, NAc; nucleus accumbens, Cb; cerebellum.

We showed the decrease of NR2A subunits in the CA1 hippocampal region after the long-term administration of both clozapine and olanzapine, but not with haloperidol. That means that in one area of the brain, antipsychotic drugs can differently influence the expression of NMDAR subunits. The difference can also be explained with the length of a period in which the antipsychotic is given. For example, in our studies, clozapine after four weeks exerted the same effect as that after six months in the study by Schmitt et al. [76]. It is not excluded that also haloperidol given longer than one month could decrease the expression of NR2A subunits in the CA1 region of the hippocampus. Tarazi et al. (2003) in their study showed results similar to ours with olanzapine—they found that after 30 days it decreases the number of NMDARs in the CA1 and CA3 regions [78]. Results from the above-mentioned studies are summarized in Figure 4. We propose that the effect of antipsychotics in the hippocampus is related to the change of composition of NMDAR subunits that cause the decrease of glutamatergic activity.

Our results are interesting when thinking about a reduction of the volume of the hippocampus in schizophrenia [79] and involvement of the hippocampus in cognitive deficits and pathogenesis of schizophrenia [78]. Much more NMDARs are located in a rat’s CA1 region, compared with CA3 [78]. In humans, the CA1 region plays a significant role in the long-term potentiation (LTP), which consists of a key molecular mechanism of memory [80]. Perhaps the antipsychotic-induced decrease of glutamatergic activity in the CA1 hippocampal region is responsible for the incomplete treatment effect of cognitive deficits in schizophrenia. Damping of glutamatergic activity in the hippocampus by antipsychotic drugs could interfere with mechanisms of learning and memorizing.

The decrease of NMDARs in the CA1 region can be explained by a direct influence of drugs on the receptor complex, although it seems that both olanzapine [78] and clozapine [19] have no affinity towards NMDARs. However, it is suggested that clozapine may modulate dopaminergic neuron activity in the rat ventral tegmental area (VTA) via binding to the NMDAR glycine sites [81]. Clozapine may also act as Mg^2+^ and voltage independent NMDAR channel blocker [82]. Haloperidol exerts an antagonistic activity against NMDARs, binding with a specific allosteric site of NR2B [83]. Although, the inhibition of NMDAR activity with haloperidol requires much higher drug concentration at the receptor, compared with regular concentrations, obtained with standard doses of haloperidol when used in the treatment of schizophrenia [83]. Thus, it seems the direct blockade of NMDARs by haloperidol has no practical importance [83].

The mechanism of action of haloperidol can be non-related to the transcription of genes. Our data indicate that haloperidol does not increase expression of NR1 subunits in the hippocampal CA1 region. It is so, although haloperidol can increase the transcription of NR1 genes in this area [84]. In our understanding, the observed decrease of NMDA expression in cell membranes is induced rather through interaction between glutamatergic, dopaminergic, and serotoninergic neurotransmission systems.

The dopaminergic and serotoninergic systems are influenced by olanzapine and clozapine, when haloperidol blocks only the dopaminergic receptors (mainly type 2, D2). Indirect evidence that the serotoninergic system is involved in NMDAR expression is the participation of the 2A type of serotoninergic receptor in the mechanism of action of PCP and ketamine. Moreover, D2 and NMDARs can be in functional antagonism [78]. Chronic D2 blocking with antipsychotics and their up-regulation decreases expression of NMDARs [85]. Through that mechanism, an antipsychotic drug can decrease excessive dopaminergic activity in the CA1 region of the hippocampus [86] and also decrease stimulation in glutamatergic projections to the limbic system and brain cortex [78]. For that reason, the changes of expression of NMDARs can be linked with a chronic blocking of 5-HT2A and D2. Moreover, the receptor blockade may induce post-translational alterations in NMDAR subunits and in this way modulate their expression in cell membranes [78].

In our experiments, clozapine and olanzapine did not influence the expression of NR2B subunit (clozapine in C1 and C3, and olanzapine in C1) as shown in Figure 2. The subunit in humans regulates NMDAR activity and its increase is associated with higher receptor activity [87]. According to Kristiansen et al. (2010), the most important NMDAR pathology in schizophrenia refers to receptors containing the NR2B subunits [55]. The NR2B subunit is involved in LTP and better synaptic transmission [88]. Decreased numbers of NR2B in receptor composition is linked to problems with learning and memory [55]. When NR2A subunits dominate in NMDAR composition, synaptic plasticity is decreased [87]. A preponderance of NR2B in NMDARs can result from its higher expression or a decrease of NR2A subunit numbers. We observed that clozapine and olanzapine decrease NR2A subunit expression in the CA1 region of the hippocampus, with no influence on a number of NR2B subunits, so clozapine and olanzapine can increase the receptor activity by changing the subunit composition in NMDARs. The lack of a significant influence of olanzapine on the expression of NR2B subunits together with the decrease of NR2A, one can explain an advantageous action of olanzapine on visual-spatial learning versus clozapine and haloperidol in schizophrenia-suffering people [89].

A similar influence of clozapine and olanzapine on the expression of NMDAR subunits shows its ability to enhance the glutamatergic activity in CA1 and improve memory mechanisms. In turn, haloperidol decreases NR2B expression in rats, as shown in Figure 2. That finding can explain how olanzapine can exert preferential influence on memory, compared with haloperidol [89].

In the gyrus dentatus, haloperidol decreases expression of NR2B subunits and olanzapine decreases expression of NR2B and NR2A subunits. The results are in accordance with Ossowska et al. (2002), who did not observe changes in the production of mRNA for NR1 subunits in the rat brain structure [84]. Change of composition of NMDAR subunits in the gyrus dentatus is an interesting finding when referred to the study by Maeda et al. (2007) [90]. Administration of NMDAR antagonist MK-801 decreases neurogenesis in mice gyrus dentatus, and clozapine but not haloperidol counteracts this effect. That means that the decrease of glutamatergic activity in the gyrus dentatus can decrease neurogenesis.

In our study, clozapine, opposite to haloperidol and olanzapine, does not change the expression of NMDAR subunits in the gyrus dentatus, as shown in Figure 2, that implies no effect of clozapine on glutamatergic activity. Haloperidol, decreasing the expression of NR2B subunits, can decrease the NMDAR activity, inhibiting neurogenesis and worsening schizophrenia symptoms in humans. Nevertheless, some studies report that long-term treatment (28 days) with haloperidol stimulated DCX-positive neuroblast formation in the rat subventricular zone (SVZ) [91] and supported adult neurogenesis in the gyrus dentatus [92].

The decrease of expression of NMDAR subunits, induced by haloperidol, can relate to functional antagonism between receptors D2 and NMDA and post-translational alterations of protein subunits of NMDARs [78,85]. The decrease of NMDAR expression can also result from the interaction between receptors D1 and NMDA. It was shown in cell cultures of striatum cells that an increase of NMDAR activity correlates with an increase of D1 receptor density. It was also proved that long-term blocking of D2 receptors decreases the expression of receptors’ D1, that in turn is a reason for the decrease in production of BDNF and reduction of neurogenesis. A conclusion is that the increase of NMDAR activity, with the increase of D1 receptor density, plays a neuroprotective role [7]. According to Maeda et al. (2007), inhibition of neurogenesis is one of the elements of complex pathogenesis of schizophrenia in humans [90].

### 3.2. Thalamus, Hypothalamus, and Neocortex

**Figure 5:**
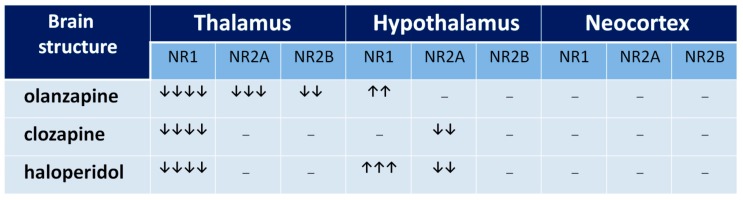
The influence of chronic neuroleptic administration on NMDA receptor subunits’ expression in thalamus, hypothalamus, and cortex in rats. Arrows indicate the subunit expression decrease or increase by 0–25% (↓), 26–50% (↓↓), 51–75% (↓↓↓), and 76–100% (↓↓↓↓).

**Figure 6:**
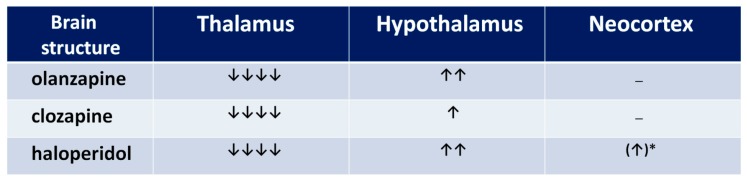
The influence of chronic neuroleptic administration on glutamatergic activity in thalamus, hypothalamus, and cortex in rats. A number of arrows indicate the neuroleptic’s potential to decrease or increase the glutamatergic activity, resulting from the summation of NR1 expression, and proportion of NR2A to NR2B expression (an arrow was added when NR2B expression prevailed, and subtracted when NR2A expression prevailed). * statistically non-significant.

**Figure 7:**
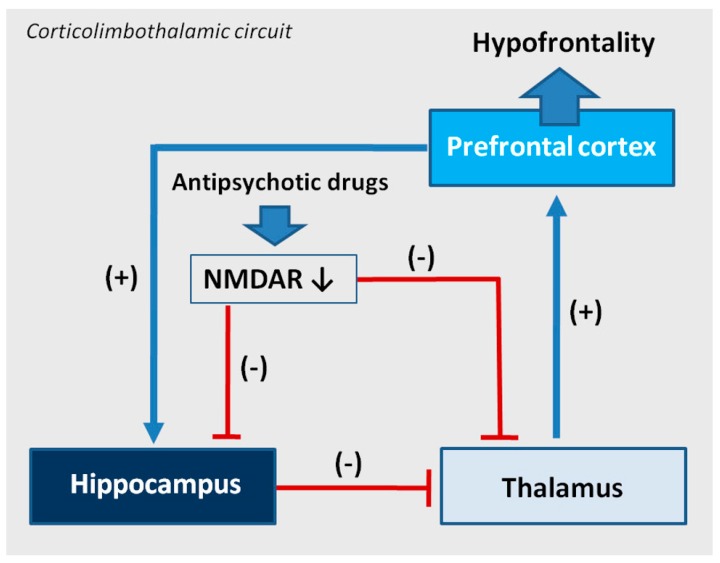
An effect of antipsychotics on cortical-subcortical circuits in the hypothetical drug-induced mechanism of hypofrontality. The prefrontal cortex sends excitatory glutamatergic outputs to the limbic system (hippocampal formation), its GABA—ergic inhibitory projections are running to the thalamus, from where glutamatergic innervation goes back to the cortex. Some hippocampal outputs target also ventral striatum, ventral pallidum, substantia nigra, and the ventral tegmental area (VTA). The activity of the corticolimbothalamic circuit can be disturbed due to antipsychotic drugs inducing a change of expression of NMDA receptors’ (NMDAR) subunits. A decrease of the number and activity of NMDARs can decrease the glutamatergic activity in the hippocampus and the thalamus and disinhibit glutamatergic stimulation from the thalamus to the prefrontal cortex, sustaining the hypofrontality.

The functional profile of the thalamus goes far beyond the classical sphere of the central sensory pathways control. This complex brain structure affects almost all aspects of higher mental processes [93,94]. Several thalamic disturbances are also present in the course of schizophrenia [95,96,97,98,99]. They are probably associated with the NMDAR hypofunction [100,101]. Targeted microinjection of NMDA inhibitors into the rat thalamus causes cognitive deficits [102], that may support this hypothesis. Olanzapine, clozapine, and haloperidol decrease NMDAR expression in the rat thalamus [67], as shown in Figure 5, suggesting that long-term treatment with antipsychotic drugs can reduce the glutamate transmission in this structure, as shown in Figure 6. There could be two consequences of that finding. The decrease of activity of NMDARs can produce a decrease of the glutamatergic activity in the thalamus, as well as inhibit an activity of the GABA-ergic interneurons, producing disinhibition in the prefrontal cortex. Both consequences can significantly impair the functioning of a corticolimbothalamic circuit, as shown in Figure 7.

Results of expression of NMDAR subunits in the hypothalamus, as shown in Figure 5, turn out to be surprising [67]. It is only the rat brain structure where we observed an increase of NR1 expression after a long-term administration of olanzapine or haloperidol. That means that the long-term action of both olanzapine and haloperidol induces the increase of the number of NMDARs in the hypothalamus. Moreover, clozapine and haloperidol, decreasing NR2A subunit expression, could also increase the NMDAR activity. Similar results were obtained by Riva et al. (1997) after three weeks of haloperidol or clozapine administration [77]. In their study, the neuroleptics decreased the expression of NR2A subunits in the hypothalamus.

Concluding, all three antipsychotic drugs can increase the glutamatergic activity in the hypothalamus. We are aware that the hypothalamus is not generally considered as the structure directly involved in the pathogenesis of cognitive dysfunction and hypofrontality. As the whole brain region, it is not a part of corticolimbothalamic loop, however it may be important in the analysis of some adverse effects of antipsychotics, e.g., neuroleptic-induced food-intake promoting action of neuropeptide Y (NPY) and orexins (OXs), which seem to depend on the hypothalamic NMDAR activity of NMDA [103,104]. Thus, an increase in the NMDAR expression as well as NPY and OXs in the hypothalamic nuclei [105], may be one of several causes of the well-known unwanted metabolic disturbances after treatment with antipsychotics.

In rat parietal cortex, we observed only a non-significant increase of NR1 NMDAR subunits. The result supports the findings of Ułas et al. (1993), who reported that after three-week administration of haloperidol, the expression of NR1 subunits was increased [106]. As a probable mechanism, the blockade of D2 receptors was proposed. Hanaoka et al. (2003) showed that haloperidol and clozapine given for two weeks decrease NR1 and NR2B subunits in rat frontal cortex [75]. In turn, clozapine administered for six months decreases the number of NR1 subunits in the dorsolateral prefrontal cortex. In another study, both haloperidol and clozapine decrease expression of NR2A in rat prefrontal cortex [76]. The discrepancies can result from the fact that different cortex areas were studied and the changes of expression of NMDAR subunits indicate a brain regional specificity of antipsychotic drugs’ influence on NMDAR composition [19].

## 4. An Antiglutamatergic Activity of Antipsychotics May Be Co-Responsible for Hypofrontality

As described, the change of composition of NMDARs can be considered the next mechanism of action of antipsychotic drugs. Chronic administration of antipsychotics changes the composition of NMDARs in a different way, depending on the brain area and the type of drug. NMDARs are richly represented on GABA-ergic interneurons and play an important role in the regulation of their activity. GABA-ergic interneurons regulate the corticolimbothalamic circuit that plays an important role in cognition. Disturbances in the circuit are suggested to explain hypofrontality in mental disorders, especially in schizophrenia. From a neurophysiological view, the corticolimbothalamic circuit is based on many anatomical structures, presented in Figure 7. Pathology taking place in those structures influences the neuronal activity in the circuit and results in hypofrontality and mental symptoms like cognitive dysfunctions and negative symptoms in schizophrenia. For that reason, antipsychotic-induced changes of expression of NMDAR subunits in the thalamus and hippocampus changing the activity of NMDARs can relate to the formation of cognitive dysfunctions and negative symptoms in schizophrenia in humans, as shown in Figure 7. The described mechanism may consist of a part of the not fully understood mechanism of hypofrontality.

As showed, studied antipsychotics inhibit NMDAR activity in the thalamus, so according to the chronic PCP model, they can disinhibit glutamatergic activity in this structure, leading to disinhibition of neurotransmission in the corticolimbothalamic circuit and in a longer perspective, in the receptor-adaptation mechanism, to hypofrontality [67]. Additionally, the decrease of glutamatergic activity in the thalamus and the hippocampus can additionally dysregulate the corticolimbothalamic circuitry. In the hippocampus, the structure belonging to the limbic system, all studied antipsychotic drugs decrease activity of NMDAR receptors, that implies a decrease of glutamatergic activity [66].

The influence of antipsychotics on NMDAR composition has a different range. Olanzapine in rat archicortex inhibits the glutamatergic activity less than haloperidol. Hypothetically, this characteristic could make this drug favorable for cognition mechanisms related to the hippocampus. It was recently reported that olanzapine but not clozapine may increase the glutamate release in the mouse prefrontal cortex via inhibition of D-aspartate oxidase (DDO) activity [107]. One may also have some hope for the new antipsychotic drug brexpiprazole, that stimulates the glutamatergic activity in the animal prefrontal cortex in a dopamine receptor D1-dependent manner [108].

We suggest that antipsychotics change the composition of NMDARs and decrease the NMDAR activity in the thalamus and hippocampus. As a consequence, the disturbances in the functioning of the corticolimbothalamic circuit occur, increasing or maintaining the present hypofrontality in patients who suffer from schizophrenia and take antipsychotic medication. Our NMDAR model of drug-induced hypofrontality suggests that chronic antipsychotic treatment of schizophrenia can dysregulate components of the corticolimbothalamic circuit and can affect negatively the cognitive functioning of patients. The hypothesis explains in part the incomplete effectiveness of antipsychotic drugs towards cognitive disturbances in schizophrenia, reviewed in [89].

The limitation, which we see, is that we did not explore all the elements of the corticolimbothalamic circuit. Our results are limited to densitometric analysis of expression of NMRA-R subunits in brain slices, processed immunohistochemically, without checking the mRNA transcription. We also did not assess the global expression of NMDAR proteins in the brain structures. We are also aware of the general limitations of translational science when animal results are tried to refer to humans’ diseases, which we do in the conclusions.

## 5. Conclusions

(1) Antipsychotic medication can decrease the activity of NMDARs throughout the change in the composition of their subunits.

(2) Antipsychotic drugs can maintain and escalate hypofrontality, decreasing NMDAR activity in the corticolimbothalamic circuit in the brain.
